# The PELskin project-part V: towards the control of the flow around aerofoils at high angle of attack using a self-activated deployable flap

**DOI:** 10.1007/s11012-016-0524-x

**Published:** 2016-09-27

**Authors:** Marco E. Rosti, Laura Kamps, Christoph Bruecker, Mohammad Omidyeganeh, Alfredo Pinelli

**Affiliations:** 1 School of Mathematics, Computer Science and Engineering, City, University of London, London, EC1V 0HB UK; 2 Institute of Mechanics and Fluid Dynamics, Technical University of Freiberg, Freiberg, Germany

**Keywords:** Passive control, Hairy flaps, Biomimetic

## Abstract

During the flight of birds, it is often possible to notice that some of the primaries and covert feathers on the upper side of the wing pop-up under critical flight conditions, such as the landing approach or when stalking their prey (see Fig. 1) . It is often conjectured that the feathers pop up plays an aerodynamic role by limiting the spread of flow separation . A combined experimental and numerical study was conducted to shed some light on the physical mechanism determining the feathers self actuation and their effective role in controlling the flow field in nominally stalled conditions. In particular, we have considered a NACA0020 aerofoil, equipped with a flexible flap at low chord Reynolds numbers. A parametric study has been conducted on the effects of the length, natural frequency, and position of the flap. A configuration with a single flap hinged on the suction side at 70 % of the chord size *c* (from the leading edge), with a length of $L=0.2c$ matching the shedding frequency of vortices at stall condition has been found to be optimum in delivering maximum aerodynamic efficiency and lift gains. Flow evolution both during a ramp-up motion (incidence angle from $\alpha _0=0$ to $\alpha _s=20^\circ$ with a reduced frequency of $k= 0.12\, U_{\infty }/c$, $U_{\infty }$ being the free stream velocity magnitude), and at a static stalled condition ($\alpha =20^\circ$) were analysed with and without the flap. A significant increase of the mean lift after a ramp-up manoeuvre is observed in presence of the flap. Stall dynamics (i.e., lift overshoot and oscillations) are altered and the simulations reveal a periodic re-generation cycle composed of a leading edge vortex that lift the flap during his passage, and an ejection generated by the relaxing of the flap in its equilibrium position. The flap movement in turns avoid the interaction between leading and trailing edge vortices when lift up and push the trailing edge vortex downstream when relaxing back. This cyclic behaviour is clearly shown by the periodic variation of the lift about the average value, and also from the periodic motion of the flap. A comparison with the experiments shows a similar but somewhat higher non-dimensional frequency of the flap oscillation. By assuming that the cycle frequency scales inversely with the boundary layer thickness, one can explain the higher frequencies observed in the experiments which were run at a Reynolds number about one order of magnitude higher than in the simulations. In addition, in experiments the periodic re-generation cycle decays after 3–4 periods ultimately leading to the full stall of the aerofoil. In contrast, the 2D simulations show that the cycle can become self-sustained without any decay when the flap parameters are accurately tuned.

## Introduction

**Fig. 1:**
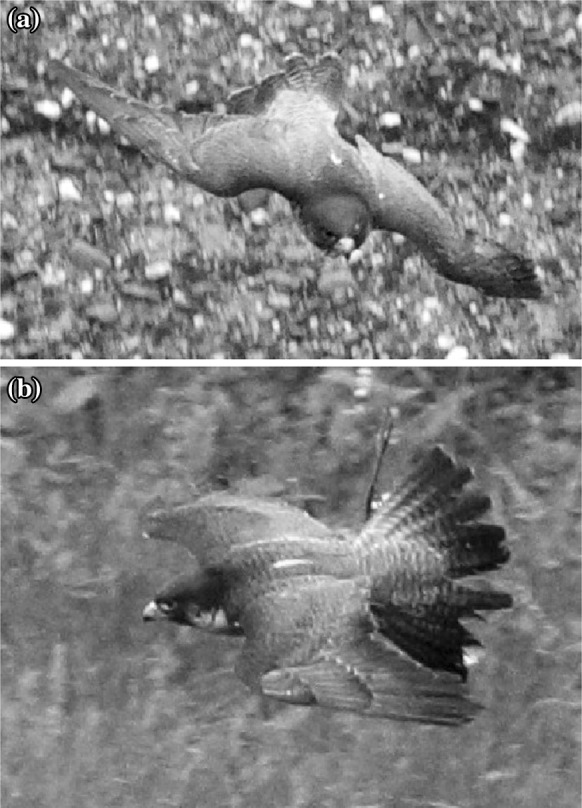
**a** Frontal and **b** side view of a falcon with popped-up feathers (taken from the measurement campaign documented in Ponitz et al. [18])

Stall is a phenomenon that arises on aerofoils at high angle of attack and is responsible of a dramatic decrease in aerodynamic performance (i.e., decrease of the lift and increase of the drag), mainly due to the flow separation on the wing surface and the appearance of large recirculating region. A stalled condition can be obtained either by keeping the angle of attack fixed beyond a certain value (static stall), or by increasing its value in time beyond the value of the static stall angle (dynamic stall, see Rosti et al. [20] for a detailed comparison between the two conditions). Recently, researchers are looking for new ways of controlling the flow separation on aerofoils at high angle of attack using devices inspired by nature. In particular, it has been observed that birds can overcome certain flight critical conditions, by popping up some of their feathers when flow separation starts to develop on the upper side of their wing [2, 3, 6] (see Fig. 1). It is believed that the feathers lift limits backflow also preventing an abrupt breakdown in lift typical of dynamic stall. With the aim of reproducing this effect, Schatz et al. [21] have shown that a self-activated spanwise flap near the trailing edge of an aerofoil can enhance lift by more than $10\,\%$ at a Reynolds number of $Re=1-2 \times 10^6$. In a similar experiment, Schluter [22] has also demonstrated that lift-breakdown is less severe when the flap is used. Wang and Schluter [24] have extended the analysis to a three dimensional wing basically confirming the aforementioned effects. Differently from the other authors, Kernstine et al. [11] found that the increase in lift can also be achieved with the flap mounted in the first half of the aerofoil, close to the leading edge. Venkataraman and Bottaro [23] performed a numerical study of the effect of hairy coatings on an NACA0012 aerofoil at low Reynolds number $Re=1100$ and high angle of attack $\alpha =70^\circ$, and found a set of coating parameters able to deliver an increase in lift (${\simeq }9\,\%$). Finally, the effectiveness of fixed versus free-moving flaps has been studied by Johnston and Gopalarathnam [9]. They found that also fixed flaps deliver an improvement in both lift and drag at high angles of attack. However, the improvements diminish when the flaps are mounted with an angle greater than of $60^\circ$.

More recently, Bruecker and Weidner [5] used hairy flaps (i.e., flaps with very small thickness) to control the dynamic stall of a wing at moderate Reynolds number $Re=77{,}000$, observing a delay of the dynamic stall. The authors claim that the delay is achieved by the reduction of the backflow, and by a regularization of the shear layer roll-up process. Moreover, they suggest that the onset of non-linear growth in the shear layer is delayed via mode-locking of the fundamental instability mode with the motion of the flaps.

Beneficial aerodynamic performance were also obtained using flexible covert mounted on a circular cylinder. Specifically, Favier et al. [7] conducted a numerical investigation into a hairy coating applied to a two-dimensional circular cylinder at a Reynolds number of $Re=200$. Their results show that the coating is able to reduce both the overall drag (by ${\simeq }15\,\%$) and the lift fluctuation (by ${\simeq }44\,\%$). Similar results were obtained at much higher Reynolds numbers in experiments involving a cylinder equipped with flexible flaps on its lee side (the flaps were not very different from the ones considered in the present study [13]). As final examples of the aerodynamic benefits that can be obtained using slender hairy appendages, it is also worth mentioning the net lift force that can be generated by using a single passive filament hinged on the rear of a bluff body (the generated lift is a consequence the wake symmetry breaking [1]) and the modifications that flexible hairy coatings can induce in near-wall turbulence [4, 10].

In the present work we have focused on the passive control of a NACA0020 aerofoil using self-adaptive flaplets mounted on the suction side. In particular, we have considered various hairy flap configurations and analysed their influence on the separated flow both at a static high angle of attack, and during a ramp-up manoeuvre. The analysis has been carried out both experimentally and using numerical simulations.

## Experimental set up and numerical formulation

### Experimental set up

In what follows, we will just provide a short summary on the experimental water tunnel setup. Readers interested in a more detailed description can find more specific information in Bruecker and Weidner [5] that considered the same conditions, except the structural properties of the attached flaplet. The aerofoil that has been considered is a NACA0020 with a chord length of $c = 0.2\,\hbox {m}$ and a span width of $0.5\,\hbox {m}$. The suction side of the aerofoils span was equipped with a thin flap (span width $s=250\,\hbox {mm}$, length $L=40\,\hbox {mm}$) hinged by the trailing edge region using an elastomeric tape (flap hinge located at $x_F/c=0.6$). The dynamic response of the flap in water, was determined using a step response test in quiescent conditions. In particular, the flap was elevated from the suction surface at an angle of $30^\circ$ and then released. The subsequent movement of the flap was recorded with a high-speed camera. The typical response time, here defined as the time required to reach $5\,\%$ of the asymptotic value, was found to be of about $250\,\hbox {ms}$ with the resulting flap motion resembling the one of a critical over damped oscillator. Note that, there is no gravity influence on the flap motion, because the wing was mounted with its spanwise axis aligned with the vertical direction in the water tunnel. Differently from the experiments reported in [5], where dense rows of slender, flexible flaplets were considered, here we have focused on a single rigid flap, extending over almost the full wingspan. The reason for considering a different flap configuration was mainly dictated by the weak flexural stability exhibited by the original configuration of [5] that did not allow to draw any clear cut conclusion on the flow-structure interaction process.

The influence of the flap on the flow around the NACA0020 aerofoil was investigated at a chord Reynolds number of $Re=U_{\infty } c/\nu = 77{,}000$ in a water channel at a bulk flow velocity of $U_\infty = 0.38\,\hbox {m/s}$. Since oscillating and subject to a ramp motion aerofoils present qualitatively similar stall processes, we have preferred to consider the second case because of the simpler requirements on the synchronization between measurement technique and aerofoil motion. The dimensions of the transparent test section in the water tunnel were $0.4\,\hbox {m}\times 0.4\,\hbox {m} \times 1.5\,\hbox {m}$ (width $\times$ height $\times$ length). The aerofoil was held vertically, top to bottom in the open measurement section. Measurements were first carried out at a constant angle of attack ($\alpha = 17.5^\circ$) using standard 2D DPIV as a reference case. Ramp-up experiments were subsequently considered. To impose the ramp-up motion, a motor placed on top of the water channel was used to turn the aerofoil at a constant rate from zero angle of attack $\alpha =0^\circ$ to the final state at $\alpha =20^\circ$ with a reduced frequency of $k = \dot{\alpha } = 0.12 U_{\infty }/c$ in the linearly growing region of the ramp function. Standard DPIV recordings were taken during the manoeuvre (camera PCO 1600, $1600\,\hbox {px} \times 1200\,\hbox {px}$ resolution, recording frequency 14 *Hz*, illumination with a pulsed Nd:YAG Continuum Minilite laser). The DPIV vector fields were processed using Dynamic Studio V2.30 (Dantec Dynamics) with an adaptive cross-correlation algorithm on a $32\,\hbox {px} \times 32\,\hbox {px}$ grid with an overlap of 75 and a peak validation algorithm. The velocity vectors were then locally smoothed using a moving average filter with a $5 \times 5$ kernel size. In addition to the PIV measurements, a high-speed camera was used to record the motion of the flap tip.

### Numerical formulation

**Fig. 2:**
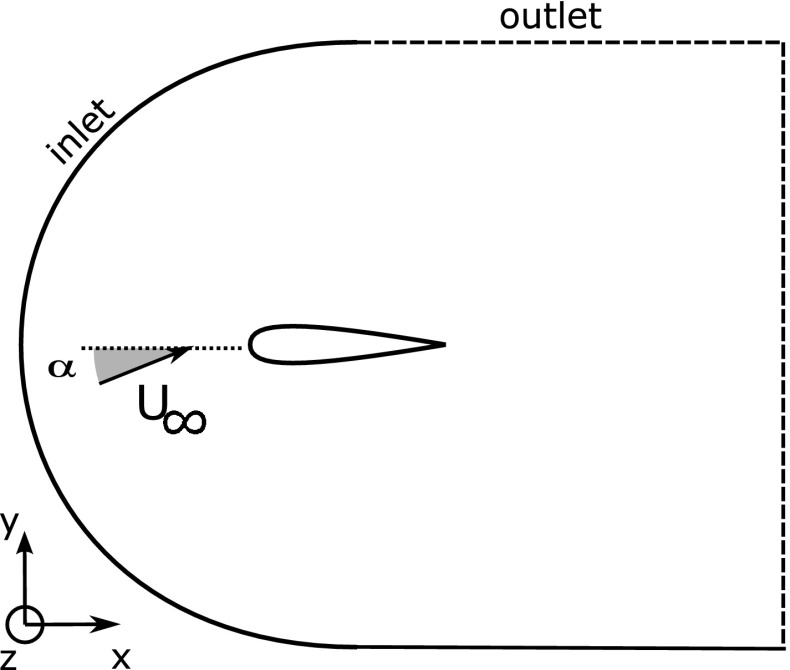
Sketch of the computational domain (geometric scaling adopted for illustrative purpose). Domain size: $-1.5c<x<5c$ and $-5c<y<5c$

The numerical simulations have been carried out using a well-established three-dimensional, time accurate incompressible Navier-Stokes solver that has been validated for a number of different flow configurations, including flows around aerofoils in both steady and unsteady ramp-up motion [20]. Here, we limit ourselves to two-dimensional incompressible unsteady flow fields around a NACA0020 aerofoil. Figure 2 shows the computational domain, with the *x* and *y* axis (also indicated with $x_1$ and $x_2$) denoting the directions parallel and normal to aerofoil chord, respectively. Also, *u* and *v* ($u_1$ and $u_2$) denote the x-wise and y-wise components of the velocity vector field. In an inertial, Cartesian frame of reference and using Einstein’s summation notation, the dimensionless momentum and mass conservation equations for an incompressible flow read as

1 $$\begin{aligned} \dfrac{\partial u_i}{\partial t} +\frac{\partial u_i u_j}{\partial x_j}= & {} -\frac{\partial P}{\partial x_i} + \frac{1}{Re} \frac{\partial ^2 u_i}{\partial x_j \partial x_j}, \end{aligned}$$

2 $$\begin{aligned} \dfrac{\partial {u_i}}{\partial x_i}&= 0, \end{aligned}$$

where $Re=U_\infty c/\nu$ is the Reynolds number based on the chord length of the aerofoil *c*, and the approaching free-stream velocity magnitude $U_\infty$ ($\nu$ being the kinematic viscosity). Unless otherwise stated, we use $U_\infty$ and *c* as the velocity and length scales for normalisation throughout the paper.

Equations (1) and (2) are discretised on a collocated grid using a finite volume formulation. In particular, the fluxes are approximated by a second-order central formulation, and the method of Rhie and Chow [19] is used to avoid spurious pressure oscillations. The equations are advanced in time by a second-order semi-implicit fractional-step procedure [12], where the implicit Crank–Nicolson scheme is used for the wall normal diffusive terms, and the explicit Adams–Bashforth scheme is employed for all the other terms. The Poisson equation that must be solved to enforce the solenoidal condition on the velocity field in the framework of a pressure correction scheme is solved using a preconditioned Krylov method (PETSc library). The code is parallelized using the domain decomposition technique and the MPI message passing library. More details on the numerical formulation and the corresponding validation campaigns can be found in [15, 16, 20].

The simulations have been carried out considering the same aerofoil as the one used in the experiments (i.e., a symmetric NACA0020 aerofoil). The computational domain ($-1.5c<x<5c$ and $-5c<y<5c$), sketched together with the body fitted C grid arrangement in Fig. 2, is bounded by an external surface encompassing both the inlet and the outlet. To determine which portion of the outer boundary is either an inlet or an outlet, at each time step the flow direction in the vicinity of the boundary is determined. If the flow points outward, the corresponding portion of the boundary is assumed to be an outlet, and is treated using a convective boundary condition. Conversely, if the flow direction is directed inward, the corresponding boundary surface is considered to be an inlet, and a Dirichlet type condition is used. The values on the Dirichlet portion are determined by considering an irrotational approximation and by solving a companion potential equation discretised via a Hess-Smith panel method [8]. When considering a ramp-up case, the Dirichlet inlet conditions are also modified in time to keep into account the change in incidence of the incoming velocity field.

An alternative formulation to impose the ramp-up manoeuvre would consist in rotating the aerofoil in time using a non-inertial frame of reference mounted on the wing [25] (axis of rotation passing through the centroid of the foil). We have evaluated the difference in the results when considering the two approaches and no significant differences between the two has been observed when low reduced frequencies are considered. In particular, the variation in the lift and drag integral values revealed to be marginal.

As far as the remaining boundary conditions are concerned, we impose: impermeability and no-slip conditions on the aerofoil wall, and continuity of the flow variables through the top and bottom planes generated by the C-grid shape downstream of the trailing edge.

All the simulations have been undertaken by fixing the chord Reynolds number to 2000 (Reynolds number effects are discussed in Sect. 3.3). The angle of attack is kept at $20^\circ$ in the static case, and varies according to a ramp function from $0^\circ$ to $20^\circ$ with a non-dimensional rate of change equal to $\dot{\alpha } = 0.12 U_\infty /c$ in the linearly growing region of the ramp function.

The grid density and nodes distribution has been tuned by undertaking a number of preliminary simulations and by carrying out a grid convergence analysis considering a coarser and a finer grid obtained by either decreasing or increasing by $30\,\%$ the number of grid points in all the directions. The grid dependency of the results has been evaluated by considering the first and second order statistics, and the comparison between the medium and finer grid showed no significant differences. Finally, we have found that a grid with $1729 \times 391$ nodes in the $x_1$ and $x_2$ direction, respectively, delivered a reasonable resolution. Further details on the code and the procedure that has been followed to generate the grid, can be found in Rosti et al. [20].

**Fig. 3:**
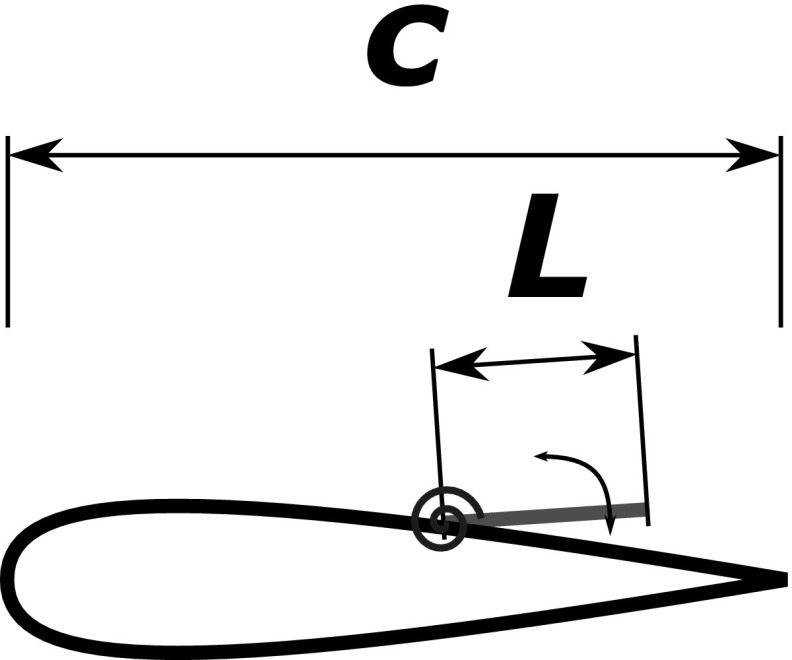
Sketch of the flap hinged on the suction side of the aerofoil through a torsional spring

The conceptual aerofoil-flap configuration that has been considered is sketched in Fig. 3 showing the NACA0020 aerofoil with the flap hinged via a torsional spring in the trailing edge region. The flap motion takes place in the *x*-*y* plane (no torsion allowed around their main axis), and is modeled using the second order ordinary differential equation

3 $$\begin{aligned} I {\ddot{\theta }} + C {\dot{\theta }} + K \theta = {\mathcal {T}} \end{aligned}$$

$\theta$ being the angular displacement, *I* the flap inertia, *C* and *K* the spring dumping factor and stiffness, and ${\mathcal {T}}$ the torque exerted by the fluid on the flap. The fluid-solid coupling of the flaps are modeled by the inclusion of a volume force $f_i$ in the momentum equation (Eq. 1), and by the torque ${\mathcal {T}}$ in (3). Both their values are determined by the immersed-boundary RKPM method [17] supposing a zero thickness flap. For further details on the coupled fluid-flow and moving flap formulations, the interested reader can refer to one of the contributions of the present special issue (*The PELskin project—part IV—Control of bluff body wakes using hairy filaments*).

## Results and discussion

### Experimental results

**Fig. 4:**
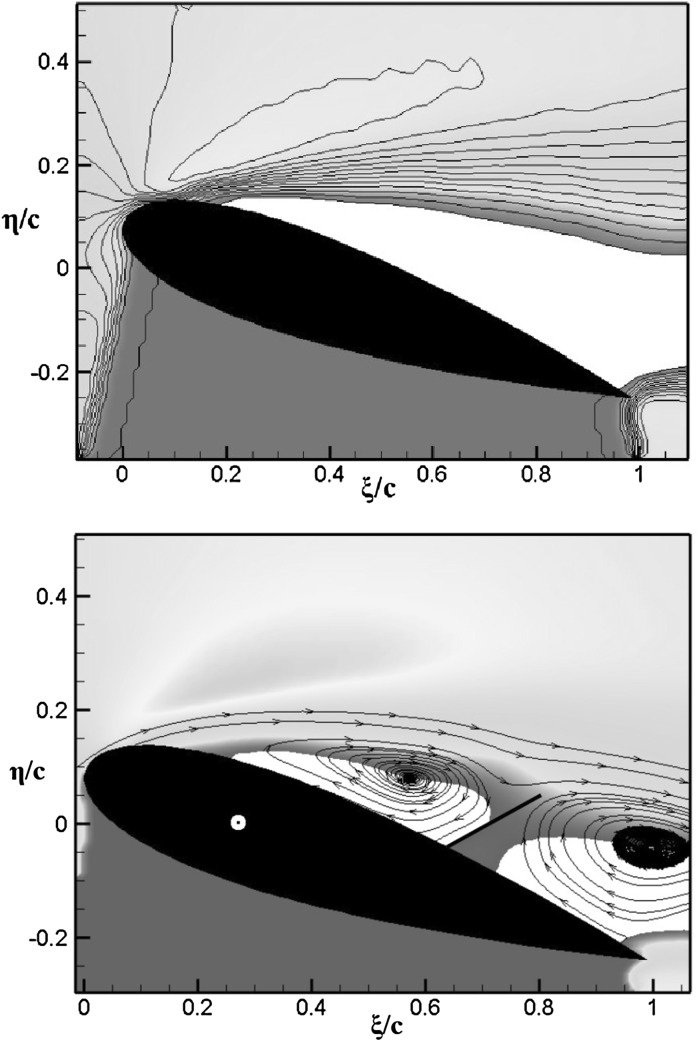
Mean streamwise velocity field around a NACA0020 at constant angle of attack of $17.5^\circ$ and $Re=77{,}000$. *Top* plain aerofoil, *bottom* aerofoil with flap. The *blue region* represents the shadow-region where the light-sheet is blocked by the model. *White color* indicate regions of negative streamwise velocity, and contour levels goes from 0 (*blue*) to $1.8 U_\infty$ (*red*). (Color figure online)

In what follows, we report the main experimental results. Firstly, we consider the static case at an angle of attack of $17.5^\circ$ corresponding to the situation in which the flow around the plain aerofoil becomes fully stalled. For both the cases with and without the flap, to determine the mean flow velocity field, given in Fig. 4, we have averaged 100 PIV snapshots.

In the plain aerofoil case the white region at the top of Fig. 4 indicates a large open backflow region that extends over most of the suction side. This region increases in size with downstream position and finally leads to a considerably large wake region downstream of the trailing edge. On the other hand, the aerofoil with the flap shows two separate regions of backflow in Fig. 4 (bottom) that are not connected and are split by the presence of the flap. The mean deployment angle of the flap is around $50^\circ$. The streamlines patterns allow to characterise the backflow regions as large recirculating vortices trapped ahead and behind the flap.

**Fig. 5:**
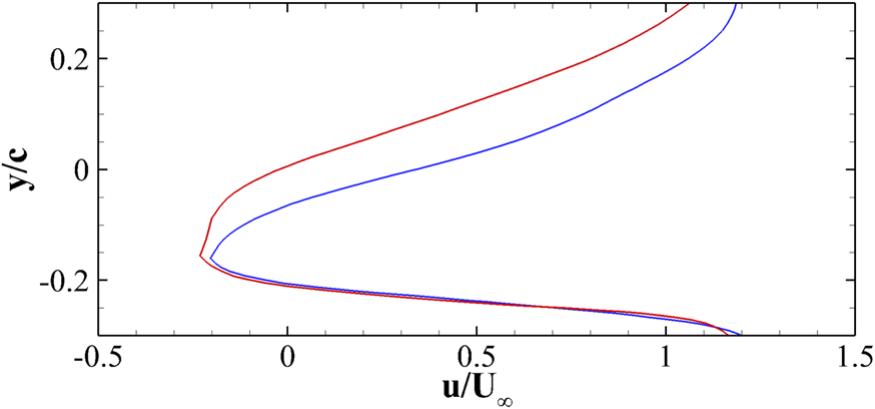
Profile of the streamwise velocity component u(y) at location $\xi =1.075c$ downstream of the trailing edge of the aerofoil. The *red* and *blue* lines are used for the cases without and with the flap, respectively. (Color figure online)

**Fig. 6:**
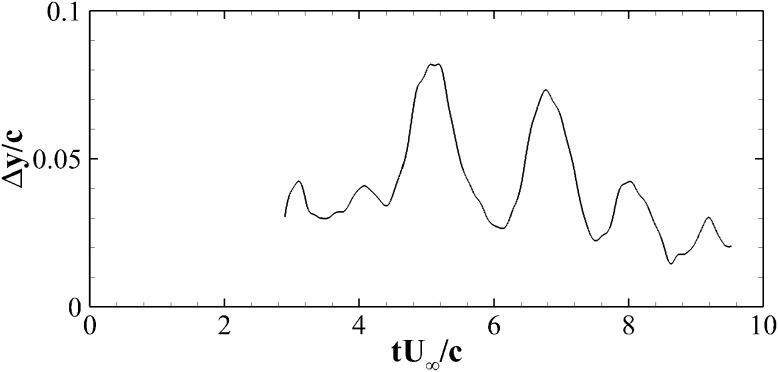
Temporal evolution of the vertical tip of the flap after ramp-up procedure. The distance is measured as $\Delta y/c$ relative to the surface of the aerofoil and is proportional to the deployment angle of the flap

A comparison of the streamwise velocity profiles between the flap and the no-flap cases taken downstream of the trailing edge is shown in Fig. 5. The figure clearly reveals a smaller wake thickness when the aerofoil with the flap is considered. As a consequence, being the wake deficit smaller than in the case of the plain aerofoil, a reduction of drag coefficient $C_D$ is expected. Thus, the presence of the flap in highly loaded conditions promotes a decrease in the aerofoil drag. Moreover, the reduction of the separated region would imply an increase in the circulation thus increasing the lift. The conjecture motivated by the PIV measurements about the increase in aerodynamic efficiency obtained with the use of the flap is further supported by the results of the numerical simulations given further below.

Before reporting the numerical results, further experimental measurements on the flap motion are briefly reviewed for the case of the single flap during a ramp-up manoeuvre. Details of the flow features and the flaps movement for the case of 3 rows of flexible flaps showing a regular roll-up of the shear layer and therefore a rather regular motion of the flaps can be found in in Bruecker and Weidner [5]. In the case of the single rigid flap, we have obtained similar results on its movement. Figure 6 displays the vertical distance of the tip of the flap from the surface of the aerofoil $\Delta y/c$ as a function of the non-dimensional time $t U_{\infty }/c$. The initial time $t=0$ is set to match the moment when the ramp-up motion is started, while the recording of the flap motion, detected with the high speed camera, starts later when the aerofoil has already reached the final angle of attack $\alpha =20^\circ$. After the completion of the ramp-up manoeuvre, a first strong peak appears corresponding to the deployment of the flap at about $t=5 c/U_\infty$. This initial peak is followed by another one at $t=6.5 c/U_\infty$ and by a third one at about $t=8 c/U_\infty$. This alternating pattern is the consequence of a periodic angular oscillation of the flap at a frequency of about $f\approx 0.66 U_\infty /c$. The movement also shows some damping as reflected in the slightly decreasing amplitude over the recording period. Due to the limited memory of the high-speed camera, a longer term evolution could not be captured. However, it seems reasonable to argue that the oscillations slowly would progressively damps out until reaching the steady time behaviour. Finally, it is remarked that in the experiments the flap width in the span does not cover the whole size of the wing. For this reason some discrepancies between the experiments and the 2D simulations, illustrated in the next section, can be anticipated. In particular, three dimensional effects at the spanwise edges of the flap may introduce additional stream wise vorticity that the 2D simulations are unable to capture.

**Table 1:** List of the cases analysed numerically

Case	$\alpha$	$f{/}f_s$	*L*/*c*	$x_F/c$	*n*
Reference	$0^\circ {-}20^\circ$, $20^{\circ }$	–	–	–	–
Optimal flap	$0^\circ {-}20^\circ$, $20^{\circ }$	1.0	0.2	0.7	1
*f* ↑	$0^\circ {-}20^\circ$	2.0	0.2	0.7	1
*f* ↓	$0^\circ {-}20^\circ$	0.5	0.2	0.7	1
*L* ↑	$0^\circ {-}20^\circ$	1.0	0.3	0.7	1
*L* ↓	$0^\circ {-}20^\circ$	1.0	0.1	0.7	1
$x_F$	$0^\circ {-}20^\circ$	1.0	0.2	0.8	1
$x_F$ ↓	$0^\circ {-}20^\circ$	1.0	0.2	0.6	1
$n$ ↑	$0^\circ {-}20^\circ$	1.0	0.2	0.6, 0.8	2

The aerofoil is NACA0020 and the chord based Reynolds number is $Re=2000$. $\alpha$ is the angle of attack (static angle or ramp-up). The flap parameters, i.e., the ratio between the spring natural frequency and the shedding frequency $f/f_0$, the flap’s length *L*, the hinge position $x_F$, and the number of flaps *n* is provided

**Fig. 7:**
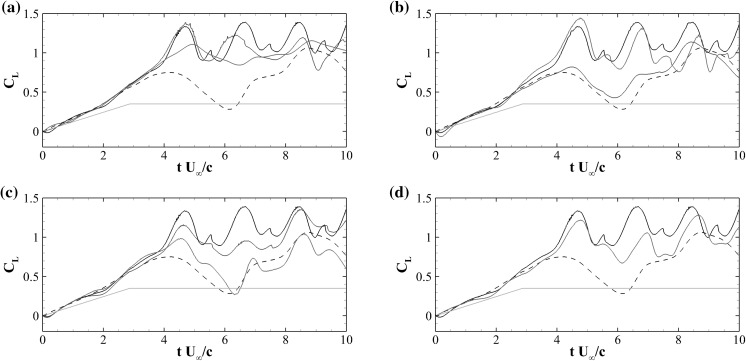
Evolution of the lift coefficient $C_L$ during a ramp-up manoeuvre. The *dashed line* is used for the clean aerofoil, while the *solid lines* for the aerofoil with flap. In each figure, the *black line* represents the case with the optimal flap, while the *blue* and *red* ones relate to the cases where the selected parameter is decreased (*downarrow*) or increased (*uparrow*), respectively (see Table 1). The parameters that have been considered are: the natural frequency of the flap *f* (**a**), the length of the flap *L* (**b**), the *x*-coordinate of the hinge $x_F$ (**c**), and the number of flap *n* (**d**). The *green line* in all the figures is the imposed time evolution of the angle of attack $\alpha$. (Color figure online)

### Numerical results

We now consider the 2*D* flow over the NACA0020 aerofoil during a ramp-up motion at $Re=2000$. In this manoeuvre, the angle of attack follows a prescribed ramp function in time with an initial linear increase followed by a plateau at steady value of the angle of incidence. In particular, the angle of attack is varied linearly from $\alpha =0^\circ$ to $\alpha =20^\circ$ with a reduced frequency of $k = 0.12 U_\infty /c$, and then the angle is kept constant at its maximum value $\alpha =20^\circ$ (see the green line in Fig. 7). The flap motion is controlled by various parameters, such as its length, inertia, position, the torsional spring stiffness and damping factor. A preliminary parametric study has been performed in order to find a quasi-optimal configuration in terms of lift and aerodynamic efficiency. The study has been performed by considering a series of simulations with different flap parameters, having as initial condition the same fully developed zero degree angle of attack flow. Table 1 details all the flap configurations that have been considered. In particular, apart from the baseline case without flap, we have analysed flap lengths in the range $L=0.1 - 0.3$, the flap positions between $x_F/c=0.6 - 0.8$ (i.e., position of the hinge measured from the leading edge), and spring stiffnesses in the range $K=0.037 - 0.600$. Note that, the stiffness can be related to the natural frequency of the spring as

4 $$\begin{aligned} K=\left( 2 \pi f \right) ^2 I, \end{aligned}$$

where *I* is the moment of inertia with respect to the rotation axis given by $I=mL^2/3$, *m* being the mass per unit spanwise length. The values chosen correspond to a natural frequency which is between half and double the shedding frequency of the baseline foil at high angle of attack ($\alpha =20^\circ$), i.e., $f_s=0.58U_\infty /c$. Finally, we have also tested a configuration with two flaps positioned in tandem on the suction side of the aerofoil. Figure 7 shows the time history of the lift coefficient during the ramp-up motion for all the considered flap configurations (solid lines), compared to the case without flap (dashed line). In the reference case without flap, the lift coefficient increases during the ramp and keeps on increasing also after having reached the maximum angle of attack ($\alpha =20^\circ$ at $t=2.875c/U_\infty$) leading to a lift overshoot as compared to the case at static angle of incidence. After having attained a maximum value at $t\approx 4c/U_\infty$, the lift starts to decrease, and slowly, non-monotonically converges to the static lift value. The described behaviour is typical of dynamic stall and has been reported by several authors in the past, see for example McCrosky [14] and Rosti et al. [20]. The time variations of lift and drag are mainly governed by the formation, evolution and final detachment of a large scale lifting vortex, usually termed as dynamic stall vortex.

**Fig. 8:**
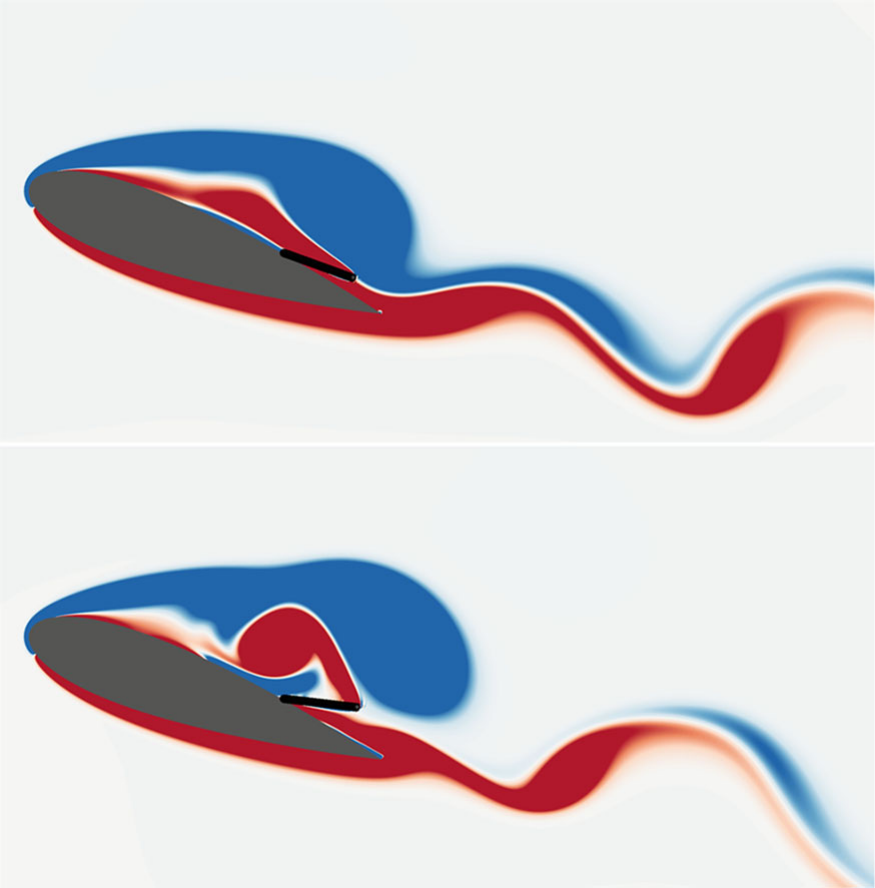
Contour of the instantaneous spanwise vorticity $\omega _z$ for the aerofoil with flap at two different time instants. *Blue* negative vorticity, red positive ($\pm 5 U_\infty /c$)

The generation, the evolution and the separation of the large stall vortex is altered by the presence of flap on the suction side of the aerofoil as reflected by the variations of the lift coefficient profiles. To determine the configuration of a flap delivering optimal aerodynamic performances we have started by considering a flap 0.2*c* long, hinged at $x_F=0.7c$, with a spring stiffness varied to produce a flap natural frequency *f* half, equal or twice the shedding frequency $f_s$ of the baseline flow at static $\alpha =20^\circ$ angle of attack (respectively indicated by a blue line, a black line and a red line in Fig. 7a). When compared to the baseline flapless case, all the flap configurations show a higher lift overshoot with much milder subsequent lift breakdowns. The optimal flap frequency is determined when the lift profile attains the highest maximum and mean values. This frequency is found to be the one that matches the shedding frequency. The continuation of the parametric campaign has been conducted by freezing the flap natural frequency to the optimal one and by varying the flap’s length (Fig. 7b). In particular, we have considered three values for the length $L=0.1c$ (blue line), $L=0.2c$ (black line, equal to experiment), and $L=0.3c$ (red line). The two longer flaps produce similar effects, with the $L=0.2c$ case having a slightly better behaviour, while the short flap is completely ineffective. Note that Eq. (4) indicates that the flap natural frequency *f* is inversely proportional to its length *L*, which optimum, i.e., $L=0.2c$, is in the order of the height of the recirculating region (see Fig. 8a) which size scales inversely with the Reynolds number.

The final analysis focused on the hinge location with the natural frequency and length of the flap frozen to the aforementioned values. Figure (Fig. 7c) reports the effect of three different hinge locations on the aerofoil lift. In particular, we have considered the following hinge locations: $x_F=0.6c$ (blue line, same as experiment), 0.7*c* (black line), and 0.8*c* (red line). The case considered in the experiment, i.e., $x_F=0.6c$, gives higher lift than the reference aerofoil, however, for the considered Reynolds number, the optimal flap location is found to be further downstream at $x_F=0.7c$. This coordinate leaves on its right a portion of the foil corresponding to the one interested by the recirculating flow measured from the trailing edge (see Fig. 8b).

**Fig. 9:**
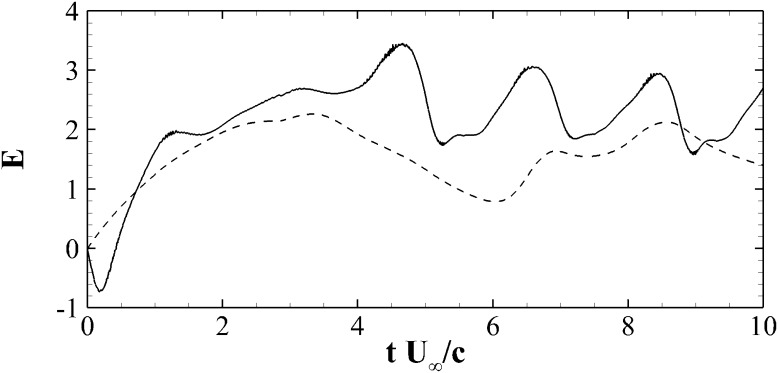
Evolution of the aerodynamic efficiency $E=C_L/C_D$ during a ramp-up manoeuvre. The *dashed line* is used for the clean aerofoil, while the *solid lines* for the aerofoil with optimal flap with $L=0.2c$, located at $x=0.7c$ and with $K=0.15$ (see Table 1

Finally, in Fig. 7d we compared the lift coefficients for an aerofoil with one flap (black) located at $x_F=0.7c$, and two flaps (red line) hinged at $x_F=0.6c$ and 0.8*c*, respectively. The increase of the number of flap does not seem to introduce any further aerodynamic benefit. Based on the results collected during the parametric campaign, we have finally decided to proceed to a further in depth analysis of the case with the flap configuration delivering the best aerodynamic performances: flap length $L=0.2c$, hinge location at $x_F=0.7$, with the natural frequency equal to the shedding frequency of the aerofoil at static $\alpha =20^\circ$ angle of attack. This configuration not only is found to increases the average lift, but also produces a dramatical increase in the aerodynamic efficiency $E=C_L/C_D$, as shown in Fig. 9.

**Fig. 10:**
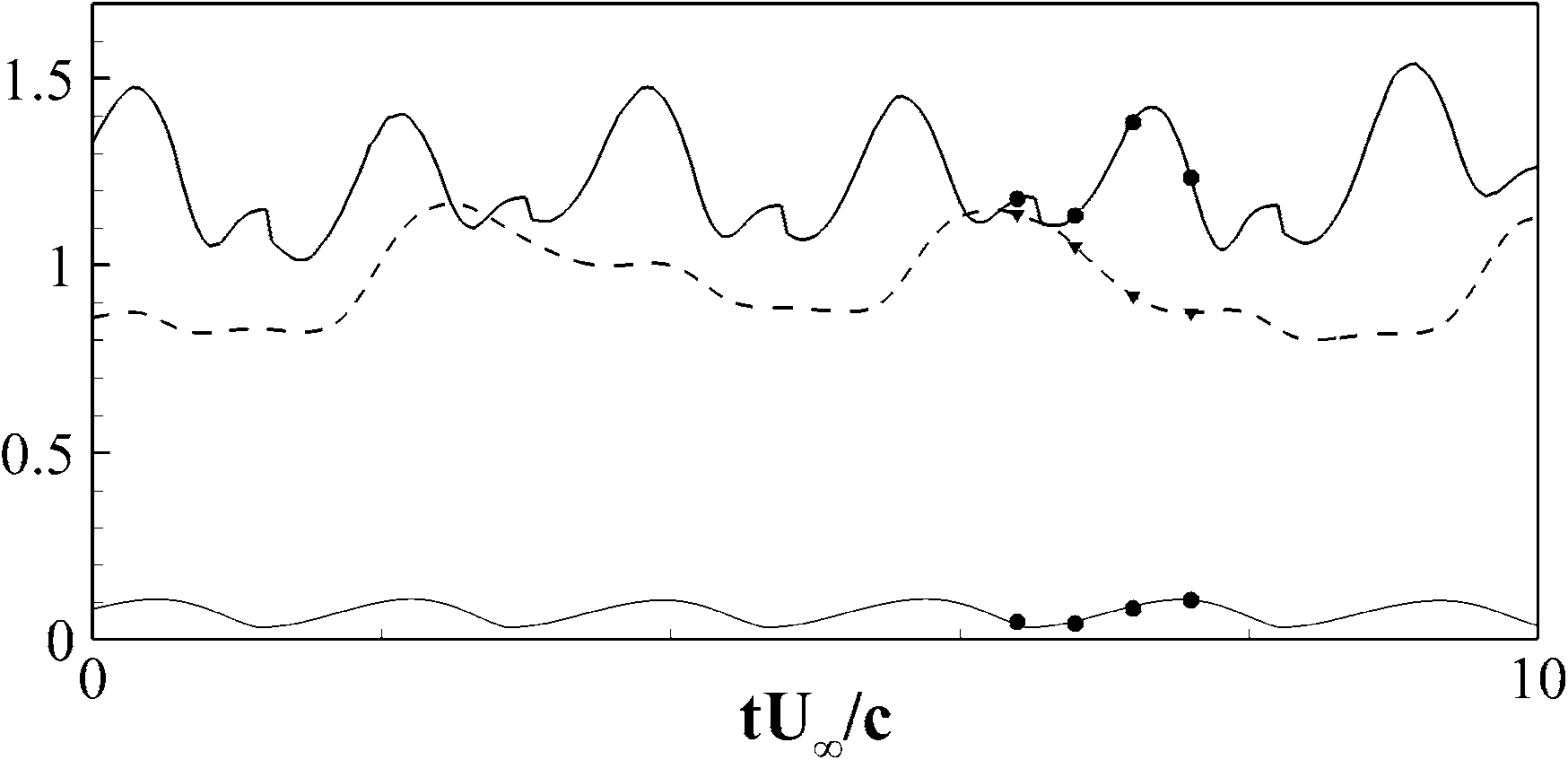
Instantaneous lift coefficient $C_L$ of an aerofoil at $20^\circ$. The *dashed line* is used for the clean aerofoil, while the *solid line* for the case with flap. The *thin solid line* represents the elevation *y* of the tip of the flap. The set of bullets on the *graphs* indicates the instants in time where the vorticity snapshots have been sampled, see Figs. 13 and 14

Next, a detailed comparison of the flow over the NACA0020 aerofoil at $Re=2000$ at a fixed angle of attack $\alpha =20^\circ$ with and without the optimal flap is considered. Figure 10 shows the instantaneous lift coefficients at $\alpha =20^\circ$. The mean $C_L$ for the clean configuration without flap (dashed line) is 1.02. When the optimal flap is used (solid line), the average lift coefficient increases to 1.28 (i.e., $38\,\%$ higher) and the mean aerodynamic efficiency raises from 1.88 to 2.2 (i.e., a net increase of $17\,\%$). The periodic lift oscillation which has a typical frequency of $f_s\approx 0.58 U_\infty /c$ strongly correlates with the flap movement (thin solid line), which shows a quasi periodic angular oscillation of the flap, similar to the one found in the experiments (see Fig. 6). In particular, the correlation coefficient between the fluctuation of the lift and the elevation of the tip of flap, has been measured to be of 0.53, with a time lag between the two signals of approximately $0.2c/U_\infty$. Finally, it is also remarked that the presence of the flap contributes in enhancing the value of the absolute value of the mean pitching moment $C_m$ (computed at the leading edge) by $22\,\%$, also strongly reducing its rms variations ($45\,\%$ less than in the flap-less case).

**Fig. 11:**
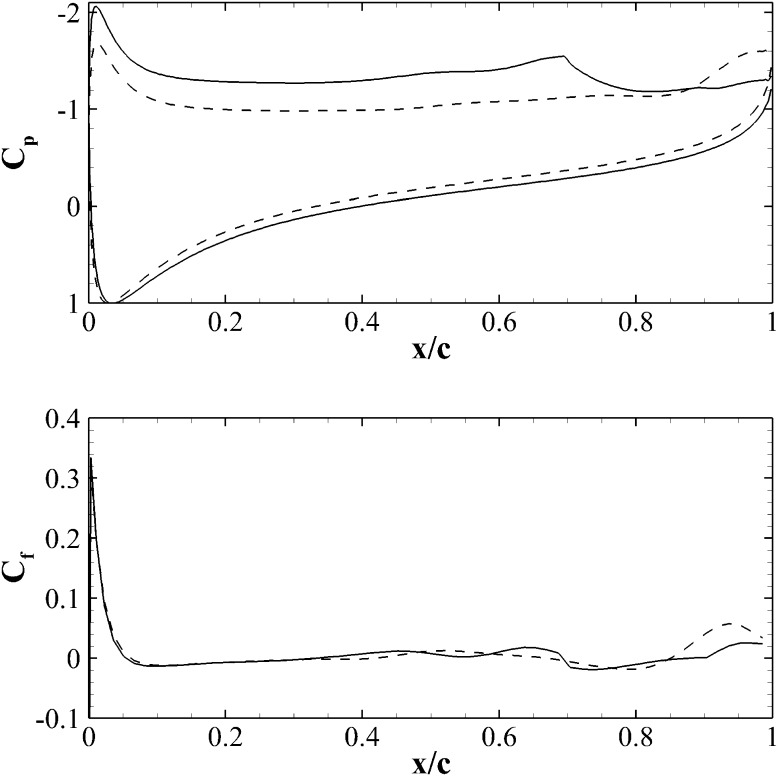
Mean pressure $C_p$ (top) and friction $C_f$ coefficients of an aerofoil at $20^\circ$. The *dashed line* is used for the clean aerofoil, while the *solid line* for the case with flap

A comparison of the mean pressure coefficients $C_p$ along the foil for the two configurations is shown in Fig. 11 (top panel). The pressure on the suction side of the aerofoil equipped with the flap, upstream of the hinge position, is lower than in the clean configuration case leading to an overall higher mean lift. It is also noticed that the value of the pressure in the suction peak is lower when the flap is considered.

Downstream of the flap hinge location, the pressure increases reaching a trailing edge value slightly higher than in the clean configuration. These results are in good agreement with the experimental results reported by Schatz et al. [21] and Bramesfeld and Maughmer [3].

The friction coefficient $C_f$ (reported in the bottom panel of Fig. 11 shows that the two aerofoils have similar friction profiles, particularly close to the leading edge where early separation occurs at $x \approx 0.06 c$. A similar information can be evinced from the experimental result provided in Fig. 4. However, the friction distribution from the mid-chord onward is slightly different in the two cases. The observed variation is probably due to the low momentum and significantly fluctuating velocities associated with the recirculation region.

**Fig. 12:**
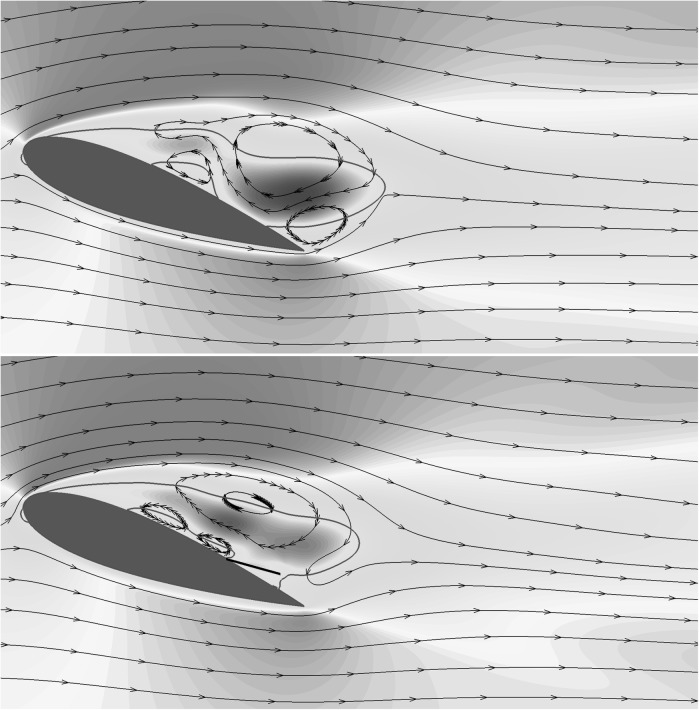
Contours of the mean streamwise velocity for the clean (*top*) and flap (*bottom*) configurations. The contour levels go from $-0.6 U_\infty$ (*blue*) to $1.4 U_\infty$ (*red*)

Figure 12 shows the iso-contours of the mean *x*-velocity component over the aerofoil in the clean configuration case (top) and when equipped with the optimal flap (bottom). The figures also incorporate the streamlines of the averaged velocity field, as well as the contour line of $u=0$ which indicates the separation bubble boundary. The latter covers almost the whole suction side, with a normal to the wall extension similar to the one recorded in the experiments (i.e., ${\approx }0.25c$). From the given results, it clearly appears that the separated regions are significantly modified by the presence of the flap. In particular, when the flap is considered the main recirculation area becomes thinner and the size of the recirculation bubble at the trailing edge is reduced too. A small, secondary recirculation bubble is present in both case, but in the case with the flap it covers a larger portion of the chord length. Differently from the experimental observations (Fig. 4), in the numerical simulations the recirculation bubble by the trailing edge is less pronounced. This difference can probably be attributed to the different modalities by which the flaps are attached to the wing. In the experiments the flap is loosely hinged on the surface, allowing a free deflection from the aerofoil surface, while in the numerical simulations the torsional spring, used at the hinge, constraints the flap movement and consequently the admissible flap elevations. Nonetheless, in both the numerical and experimental set up the use of the flap diminishes the extent of the separated region with beneficial effects on the force coefficients.

**Fig. 13:**
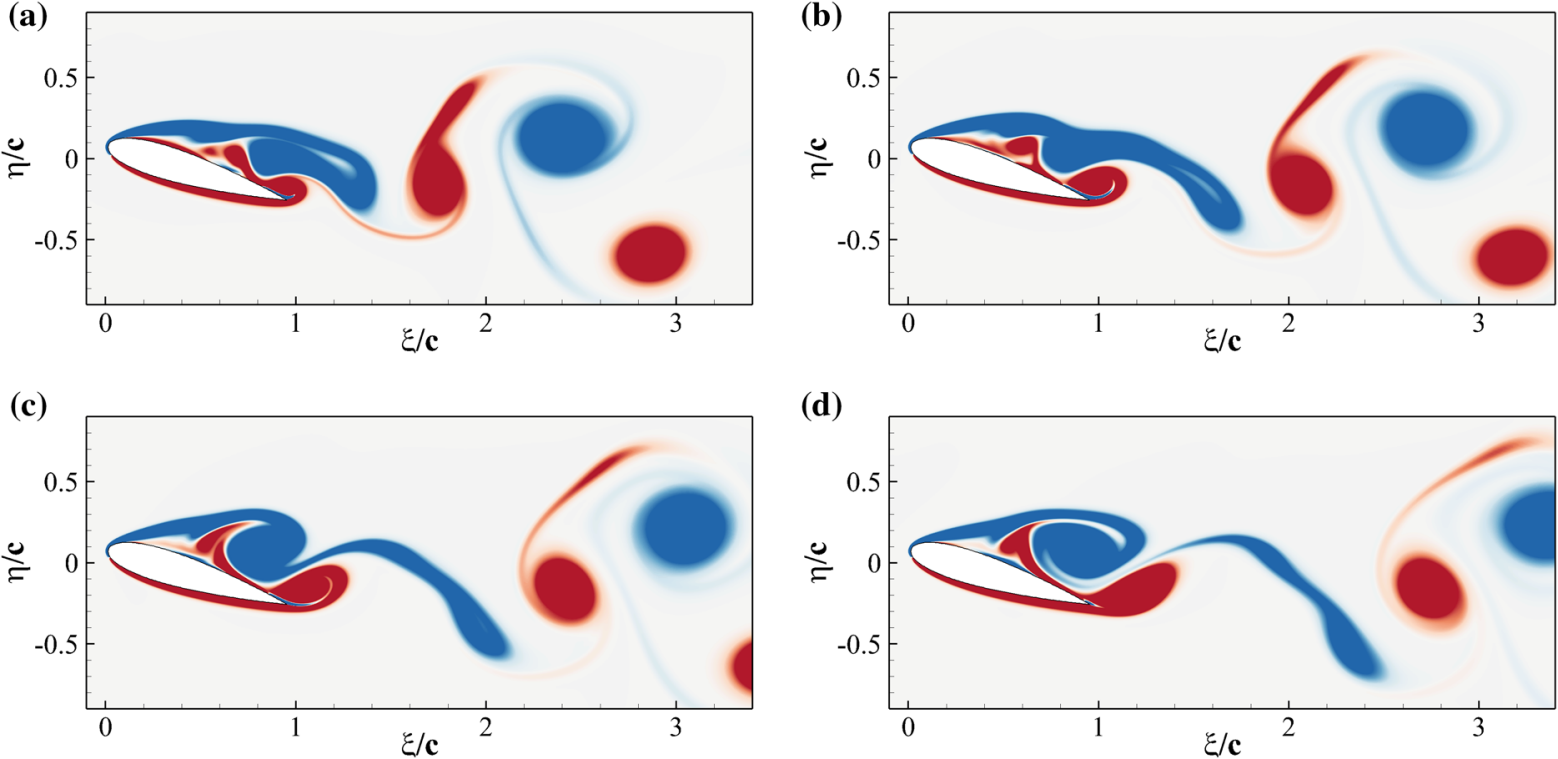
Contours of the instantaneous spanwise vorticity component $\omega _z$ during a shedding cycle (period of $1.72c/U_\infty$ non-dimensional time units). The snapshots correspond to the time instants marked in Fig. 10. Blue negative (clockwise) vorticity, red positive (counter clockwise) chosen in the range $\pm 5U_\infty /c$

**Fig. 14:**
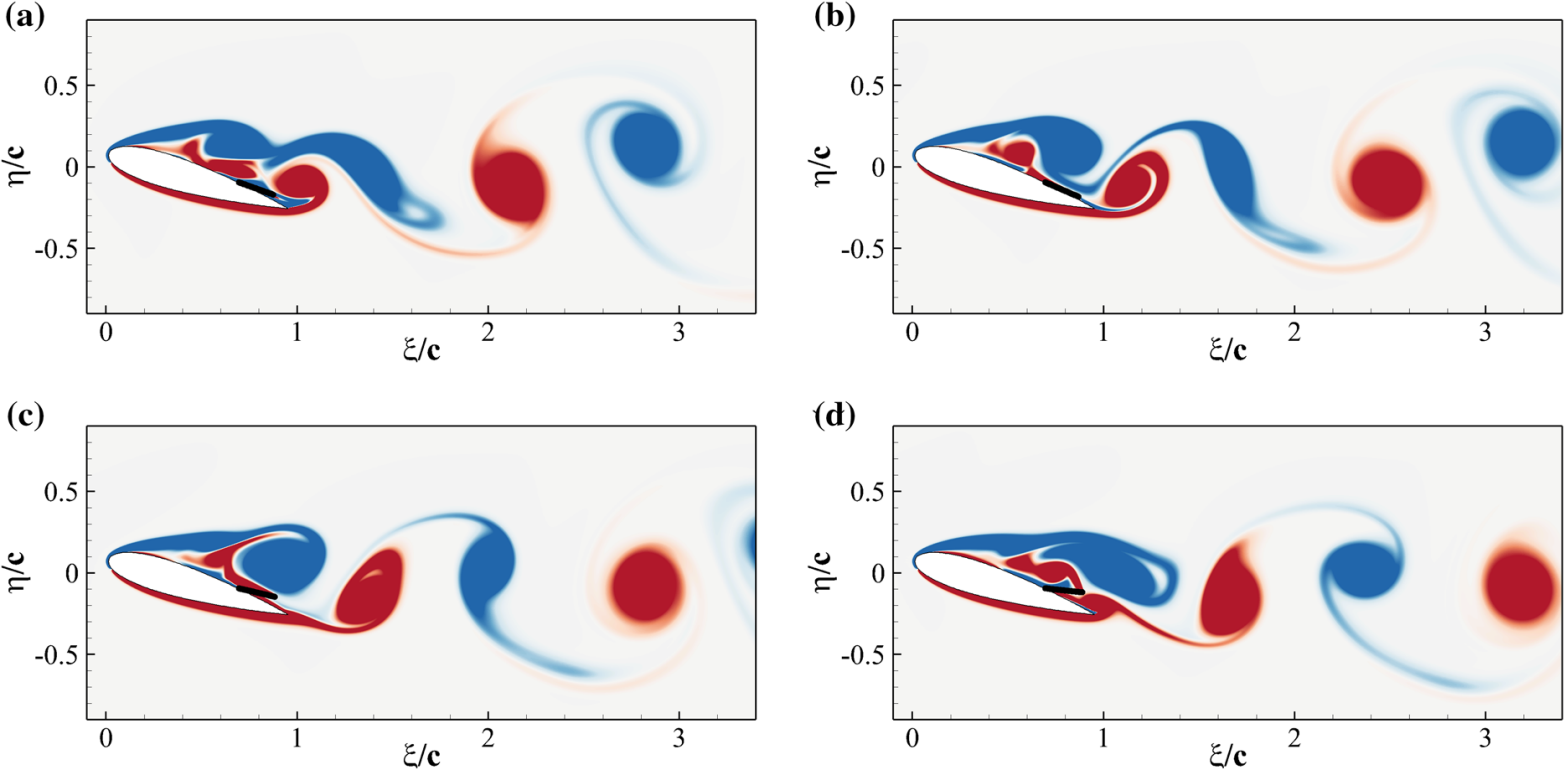
Same as Fig. 13 but with the optimal flap

To understand how the flap movement contributes to the lift generation, we now consider the instantaneous spanwise vorticity field $\omega _z$ for the case without (Fig. 13) and with flap (Fig. 14), over one shedding cycle ($T=1/f_s=1.72c/U_\infty$). The presence of two dominant vortices formed as a consequence of the leading and trailing edge shear layer instabilities characterise the time series [20]. In particular, their opposite circulations are responsible for the lift and down-force generated by the clockwise rotating vortex (blue), and the counter clockwise rotating one (red), respectively. The first few snapshots of the vorticity time series for the reference case (Fig. 13) correspond to a condition of maximum lift in which the leading edge vortex has already formed while the trailing edge one is rolling up, on the verge of being shed from the aerofoil (Fig. 13a–e). The roll up of the trailing edge vortex, is the responsible for the lift decrease that gradually recovers as the vortex is shed into the wake. A similar process takes place in the case with flap, shown in Fig. 14. In the first two snapshots (Fig. 14a, b), with the flap almost laying on the aerofoil surface, a vortex detaches from the trailing edge. Subsequently, (Fig. 14c, d) the flap reaches its maximum elevation as a consequence of the large lifting vortex that has formed above the aerofoil also inducing a maximum in the lift force. The cycle is closed by the formation of a new trailing edge vortex. In the case with flap, the vortex generated at the trailing edge is displaced downstream by the jet generated by the movement of the flap returning to its equilibrium position. The displacement of the trailing edge vortex has a two fold effect: it allows the lifting vortex to grow more and reduces the downward lift that has a negative impact on the average lift coefficient.

### Reynolds number effect

**Fig. 15:**
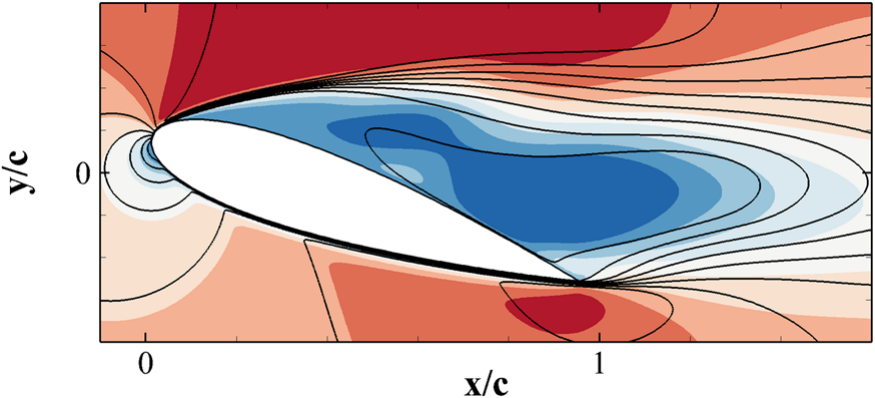
Contours of the mean flow stream-component velocity *u*. The colour contour is used for the 2*D* case at $Re=2000$, and goes from $-0.1U_\infty$ (*blue*) to $1.2U_\infty$ (*red*), while the contour lines (with the same levels) is used for the 3*D* case at $Re=20{,}000$. (Color figure online)

**Fig. 16:**
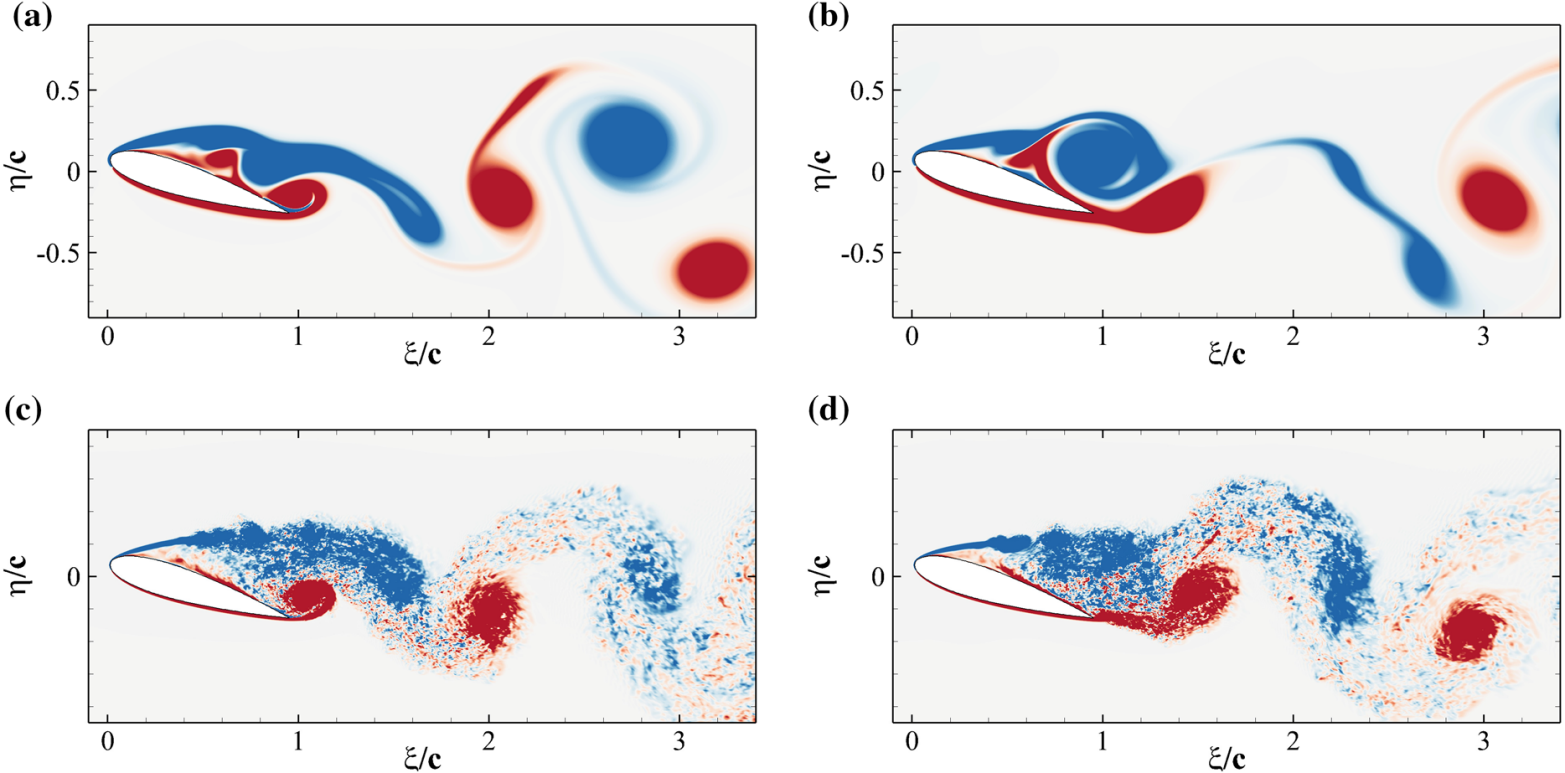
Contours of the instantaneous spanwise vorticity $\omega _z$ for the 2D (*top*) and 3D (*bottom*) cases. *Blue negative* (clockwise) vorticity, *red positive* (counter clockwise) in the range $\pm 5U_\infty /c$

We finalise by providing a comparison between the flow over a NACA0020 at $Re=2000$ (2D) and at $Re=20{,}000$ (3D, spanwise domain size: $L_z=0.9c$). This exercise is meant both to justify the parametric campaign on the flap characteristics (that has been undertaken by considering a low Reynolds number case in 2D), and to explain the similarities found between the experimental and the numerical results. The detailed numerical setup of the 3D simulation will be omitted here but the interested reader can refer to Rosti et al. [20]. Figure 15 compares the character of the mean three dimensional stream-wise velocity field at $Re=20{,}000$ and $\alpha =20^\circ$ with the two-dimensional field obtained at the same angle of attack but at $Re=2000$. The two velocity fields show similar qualitative features: large recirculating regions of comparable magnitude covering the whole suction side of the aerofoil. The unsteadiness of both the 2D and the 3D stalled cases is mainly determined by the presence, the interaction and the shedding of the two large counter rotating vortices that characterise the region above the aerofoil (see Fig. 16) and Rosti et al. [20]). The dynamic of these two large vortices governing the lift oscillations, is mainly of 2D, 7laminar nature and basically involves only the interaction of the very large coherent structures of the flow.

## Conclusion

We have considered the aerodynamic effects of a thin flap mounted on the suction side of a NACA0020 foil. The investigation has been carried out both experimentally and numerically considering a different set-up that however shared the same basic geometrical features. Despite the dissimilarities between the experimental and the numerical conditions, the two studies lead to results with a good qualitative agreement. In particular, albeit the large difference in Reynolds numbers, both approaches reveal a beneficial effect of the flap in terms of increased lift and efficiency when high angles of attack are considered. The optimum configuration (the one that delivers the highest instantaneous and mean lift coefficients) was found to consist of a single flap with length of $L=0.2c$, positioned at $x_F/c=0.6-0.7$, measured from the leading edge. This configuration has been determined using a number of numerical simulations spanning a range of flap parameters. Apart from the mentioned geometrical properties, it has also been found that to obtain a significant response to the geometrical variations, the natural frequency of the flap (that can be tuned using the torsional spring stiffness) need to be tuned to fit the vortex shedding frequency at static stall angle of attack. This numerical outcome confirms the hypothesis of the necessity of a flap-wake mode-locking to maximise the aerodynamic benefits of an elastically mounted flap as initially put forward by the experiments of [5]. When the aforementioned optimal condition is met, the simulations reveal a periodic oscillation of the lift force around a mean value higher than in the clean configuration without flap. It is also found that the mutual interaction of the flow field with the movement of flap has a strong impact on the shedding process and therefore with the structure of the wake, as manifested by high value of the correlation between the lift coefficient and the flap elevation.

A similar behaviour, characterised by a periodic oscillations of the flap, was also observed in the experiments albeit with a slightly higher frequency. A possible explanation of this difference in non-dimensional frequency, can be attributed to the larger Reynolds-number of the experiments. Indeed, the periodic nature of the flow is expected to scale inversely with the boundary layer thickness. Therefore, the frequency would increase with the value of the Reynolds numbers.
